# Planarian RNAi knockdown: feeding once might just be enough

**DOI:** 10.3389/fnins.2025.1546196

**Published:** 2025-04-30

**Authors:** Guillaume Reho, Yannick Menger, Vincent Lelièvre, Hervé Cadiou

**Affiliations:** ^1^UPR 3212, Institut des Neurosciences Cellulaires et Intégratives, Centre National de la Recherche Scientifique, Université de Strasbourg, Strasbourg, France; ^2^UMR 7364, Laboratoire des Neurosciences Cognitives et Adaptatives, Centre National de la Recherche Scientifique, Université de Strasbourg, Strasbourg, France

**Keywords:** planaria, nociception, RNA interference, TRPA1, refinement, knockdown (KD)

## Abstract

RNA interference (RNAi) is a powerful tool to knock down the expression of genes of interests. In planarians, a popular animal model to study development and regeneration processes, RNAi is easily set up by feeding the animals double-stranded RNA (dsRNA). However, there is no consensus in the literature on the amount of dsRNA needed to efficiently knock down gene expression, nor on the lasting effect of this knockdown. Here, we exposed the worms to two RNAi protocols, either feeding them dsRNA only once or three times in the span of a week. To observe the gradual loss and retrieval of nociceptive phenotypes, we exposed the worms to Allyl Isothiocyanate (AITC), an irritant and TRPA1 receptor agonist, while we knocked down the expression of the TRPA1 receptor and performed behavioral assessments over 11 weeks. We showed that feeding planarians once was sufficient to induce similar phenotypes as feeding them three times, that also lasted as long. These insights are useful to refine RNAi protocol timelines and may save some valuable resources.

## 1 Introduction

**Figure 1:**
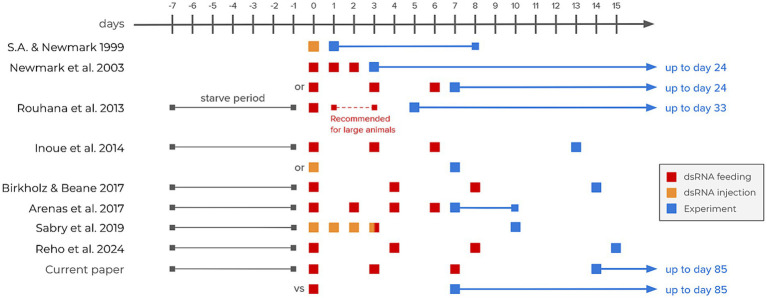
RNAi interference timelines in selected articles from the literature. The three earliest articles represent the foundations of RNAi methodology in planarians (Sánchez Alvarado and Newmark, 1999; Newmark et al., 2003; Rouhana et al., 2013). The remaining articles all focus on TRP receptors (Inoue et al., 2014; Arenas et al., 2017; Birkholz and Beane, 2017; Sabry et al., 2019; Reho et al., 2024).

The planarian animal model has shown increasing popularity in the last few decades. Known for their regenerative capabilities (Ivankovic et al., 2019), planarians have also been increasingly popular for pharmacological (Pagán, 2017) and toxicological assays (Hagstrom et al., 2015; Wu and Li, 2018). In most animal models, the technique of choice to study specific genes is the traditional gene knockout. While transgenic techniques (DNA randomly inserted into the genome) are challenging and rarely used in planarians, endogenous gene targeting to create a knockout (using homologous recombination) is even more troublesome in such asexual species reproducing through fission and regeneration (Hall et al., 2022). Fortunately, using RNA interference (RNAi) techniques to induce knockdowns is made very easy on this animal model. By either injecting or feeding planarians double-stranded RNA (dsRNA) targeting a gene of interest’s mRNA, it is possible to efficiently knockdown any protein in the whole animal with long-lasting effects (Rouhana et al., 2013). Compared to injecting dsRNA, the feeding method has the advantage of being non-invasive, and thus limits the risks of damaging tissues and inducing unwanted regenerative processes. However, there seems to be no consensus in the literature on the amount of dsRNA needed to efficiently reduce protein synthesis, nor on the lasting effect of this knockdown (Figure 1). Various articles involved feeding or injecting dsRNA three times. However, this sequence of feeding seemed to serve a precautionary purpose, lacking clear justification. We therefore tried to trace this choice to its origins. The earliest planarian RNAi methodology dates back to 1999 (Sánchez Alvarado and Newmark, 1999). In this article, Sánchez Alvarado and Newmark micro-injected dsRNA only once in planarians fragments before carrying out whole-mount immunofluorescence. In 2003, Newmark et al. showed that feeding dsRNA-expressing bacteria to whole planarians was also efficient. They wrote that “preliminary experiments suggested that the most consistent inhibition was observed after three feedings” without showing any evidence (Newmark et al., 2003). In 2013, Rouhana et al. suggested that the synthesis of dsRNA could be more easily done *in vitro* by polymerase chain reaction (PCR) rather than *in vivo* by bacteria. They also recommended “multiple dsRNA treatments for large animals and for gene knockdowns with late phenotypic manifestations” (Rouhana et al., 2013). They indeed tested this claim using functional assessments throughout 1 month after 1, 2 or 3 feedings. As they knocked down the gene *Argonaute-2*, which is regulating crucial RNA silencing processes through the RNAi-induced silencing complex (RISC), their functional assessment was the survival rate of the animals. However, since *Argonaute-2* expresses a protein implicated in the mRNA knockdown process itself, we might argue that the knockdown of components involved in the mRNA silencing process itself induces a negative feedback loop. This could slow down the RNAi process once the argonaute proteins are degraded, before it becomes fatal to the animal. Nonetheless, these three articles created the foundation of most planarian RNAi protocols used to this day, but they might be lacking functional assessment through non-lethal phenotypes. Since then, various research papers have used RNAi protocols to abolish specific behaviors in planarians, especially related to nociception through the Transient Receptor Potential Ankyrin 1 (TRPA1). Most of them kept using three dsRNA feedings within a duration of approximately a week (Inoue et al., 2014; Arenas et al., 2017; Birkholz and Beane, 2017; Sabry et al., 2019; Reho et al., 2024; Figure 1). The aim of the present study is therefore to optimize the standard planarian RNAi knockdown protocol on a gene responsible for fine-tuned behavioral responses, thus leading the way for finer gene function studies and reducing cost, resources and animals’ use.

## 2 Methods

The present methodology is similar to our previous works (see Reho et al., 2024 for more information).

### 2.1 Animals

The planarians species used in this study were *Girardia dorotocephala* (*Gd*). They were maintained in Volvic mineral water (Volvic, France) at constant 21°C and exposed to a 12 h:12 h light–dark cycle. They were fed beef liver; their water was changed and their container cleaned once a week. Worms were starved for 7 days prior to experimental testing. They were tested at least 2 h after the onset of the dark phase (2 PM). For long-running experiments, the worms were tested once every 2 weeks and fed beef liver once every other week.

### 2.2 Chemicals

Allyl isothiocyanate (AITC, CAS: 57–06-7, Sigma-Aldrich) was the main nociceptive-inducing chemical used in this study at a concentration of 50 μM. AITC is a potent TRPA1 agonist already used as a ‘scrunching inducer’ in various planarian studies (Reho et al., 2022). AITC oil was mixed with Dimethyl sulfoxide (DMSO, CAS:37–38-5, Sigma-Aldrich) at a 1:50 ratio to allow final solubilization in mineral water. The final concentration of DMSO (0.025%) does not alter the worms’ behavior (Pagán et al., 2006; Sabry et al., 2019).

### 2.3 Chemical nociceptive test

Nociceptive behavioral tests were done in an “open field” 14.5 cm diameter glass petri dish filled with 50 mL of solution (either plain Volvic water or with 50 μM AITC). Worms were tested in these open fields individually for 5 min each. Tests were performed inside a light-tight home-made chamber to ensure complete visible darkness during the tests. An infrared (850 nm) light strip and an infrared camera (Arducam IMX477) were positioned above the petri dish to record the worms at 10 frames per second.

### 2.4 Behavioral analysis

Behavioral analysis was performed on the video recordings. We assessed manually and visually, throughout the whole recordings, how the worms behave: either as a gliding movement, a scrunching movement, or anything else (namely ‘others’). We removed from further analysis worms that displayed more than 50% of ‘other’ behaviors, which was uncommon, as an exclusion criterion. Additionally, the first minute of recording out of the five was then removed from further analysis because the worms mainly displayed ‘other’ behaviors in the first minute of AITC exposure. See Reho et al. (2024) for more insight about these decisions.

### 2.5 Statistical analysis and graphical plotting

All graphical representations and statistical analysis were done using the R ‘ggpubr’ and ‘tidyverse’ packages in Rstudio (Wickham et al., 2019; RStudio Team, 2020; Kassambara, 2023). Results were expressed as average ± SEM, and n represents the number of animals in each condition. Unless stated otherwise, all statistical tests realized were pairwise Wilcoxon tests. Statistical significance was set to *p* < 5% (*), *p* < 1% (**) and *p* < 0.1% (***).

### 2.6 RNA interference

Planarians were starved for 7 days before being fed with pellets of beef liver paste containing *Gd*-TRPA1 or GFP dsRNA (0.5 μg/μL), agarose (0.3%) and blue food coloring dye (3%), as described previously (Rouhana et al., 2013; Shibata and Agata, 2018; Reho et al., 2024). Groups of 12 worms received one pellet, either only once or three times at 3 to 4-day intervals. Only animals that appeared completely blue after the pellet ingestion were kept for further investigation, as we assumed that the dsRNA quantity they ingested reached saturation of its potential effects. Their behavior was then tested every other week. Total RNA was extracted from whole animals (*n* = 5–8 worms per time point) and RT-qPCR was performed as previously described (Reho et al., 2024). The cDNA sequences from *Gd* (TR25446 for TRPA1, TR119786 for GAPDH and TR31191 for Elongation factor 2) were obtained from the annotated RNA-Seq sequence published by (Rouhana, 2017). Gene identity was confirmed by blasting the corresponding cDNA sequence with NCBI’s blast tool. Primers were designed using the Primer3 software (Table 1). In addition, we included a third highly conserved housekeeping gene (HKG) using 18S rRNA primers initially designed for *Dugesia japonica* (Yuwen et al., 2011) and that matched a highly conserved sequence between all planarian species. All primer sets were designed to achieve a high specificity (PCR efficiency >97%) and selectivity assessed by standard and melting curves, respectively. We used the web-based tool RefFinder (Xie et al., 2023), which combines the algorithms of NormFinder (Andersen et al., 2004), GeNorm (Vandesompele et al., 2002), BestKeeper (Pfaffl et al., 2004), and comparative Δ-Ct method (Silver et al., 2006) to determine the most stably expressed HKG. All samples have been run in duplicates and relative gene expression was calculated using GAPDH, the best candidate reference gene identified by RefFinder, to normalize *Gd*-TRPA1 expression according to the 2-ΔΔCt method.

**Table 1:** Primer sequences and PCR product sizes.

Primer	Sequence	PCR product size
Gd-TRPA1-qPCR-Fw	TGCATATTGTCGACGAAGGGG	115 bp
Gd-TRPA1-qPCR-Rev	TGTCCTCGGCTACCTTCAGT	
Gd-GAPDH-qPCR-Fw	TGTCTCGCTCCAATGGCAAA	119 bp
Gd-GAPDH-qPCR-Rev	AGTTTCTGCGACGGACCATC	
Gd-EF2-qPCR-Fw	ACTCGAGCCAGTTTATCAATGTG	117 bp
Gd-EF2-qPCR-Rev	GTACCGGCCACTTGAGTCTC	
Gd-18S-qPCR-Fw	AACGGCTACCACATCC	121 bp
Gd-18S-qPCR-Rev	ACCAGACTTGCCCTCC	

## 3 Results

### 3.1 Behavior (3x feeding)

**Figure 2:**
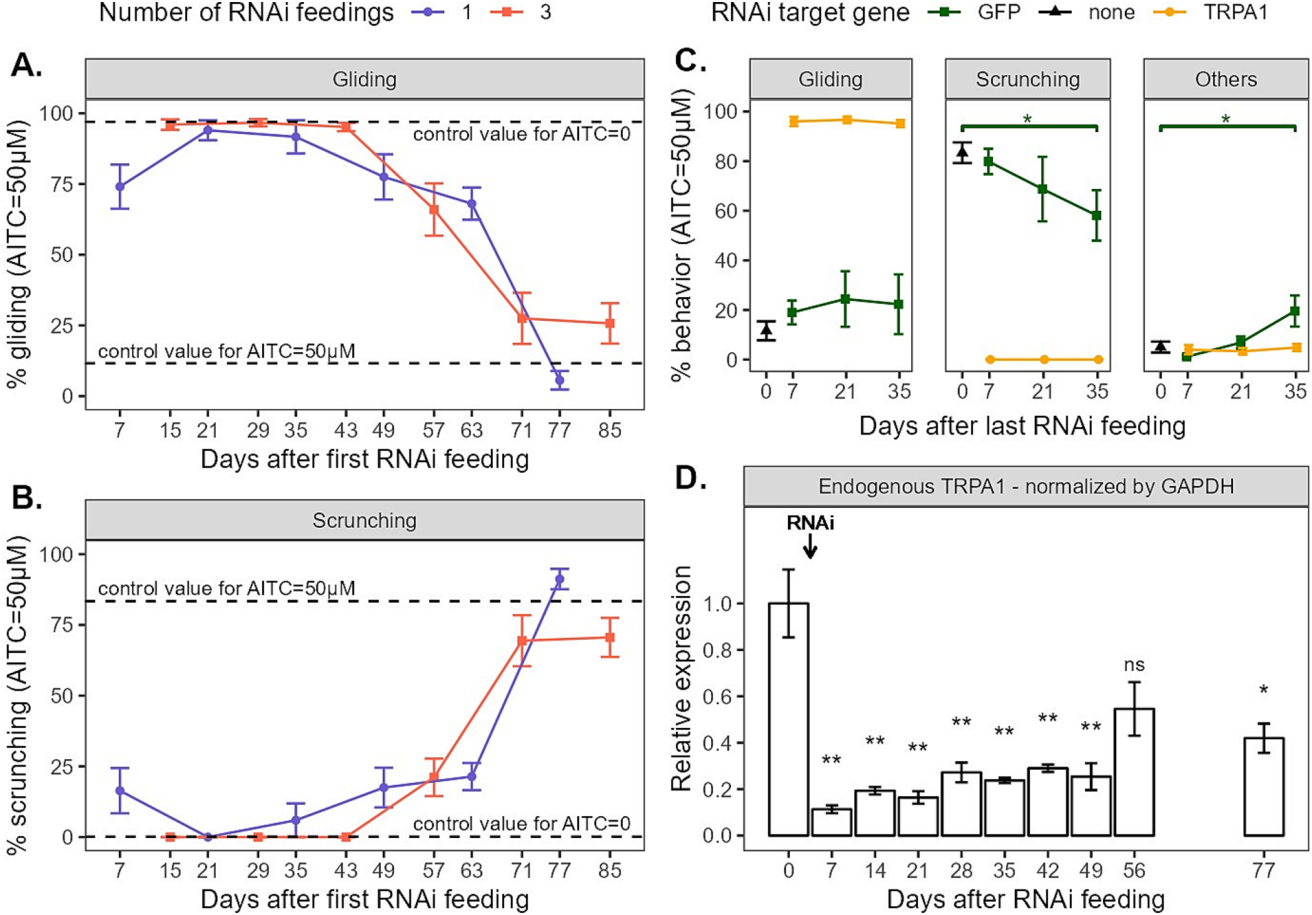
**(A,B)** Gliding and scrunching behaviors of planarians observed when exposed to 50 μM of AITC for 5 min every other week after being fed *Gd*-TRPA1 dsRNA. Dots represent the mean, and error bars represent the SEM. Dashed lines represent the mean value for untreated worms exposed to either no AITC (plain mineral water) or 50 μM AITC. **(C)** Behaviors of planarians observed when exposed to 50 μM of AITC for 5 min every other week after being fed 3 times either *Gd*-TRPA1 dsRNA or GFP dsRNA (as a control for the treatment). Untreated worms exposed to 50 μM were also included as a baseline. **(D)** Relative endogenous *Gd*-TRPA1 expression normalized by GAPDH throughout the weeks in worms being fed *Gd*-TRPA1 dsRNA once at day 0.

Worms fed TRPA1 dsRNA three times and exposed to 50 μM of AITC displayed no signs of nociceptive behavior for more than a month: they match the behavior of worms exposed to no AITC at all (Figures 2A,B). Indeed, planarians in plain water mainly glide (95.8 ± 2.1%, *n* = 6), and 3x fed planarians in AITC display significantly similar gliding behaviors at day 15 (96.0 ± 1.9%, *n* = 17, *p* = 0.55), day 29 (96.7 ± 1.3%, *n* = 11, *p* = 0.92) and day 43 (95.1 ± 1.5%, *n* = 12, *p* = 0.73). The same is true for scrunching behaviors. In plain water, planarians did not scrunch (0.0 ± 0.0%, *n* = 6) and 3x fed planarians in AITC displayed no signs of scrunching at day 15 (0.0 ± 0.0%, *n* = 17), day 29 (0.0 ± 0.0%, *n* = 11) or day 43 (0.0 ± 0.0%, *n* = 12) neither. From day 57, gliding behaviors were significantly lower than the no-AITC control values (66.0 ± 9.2%, *n* = 9, *p* < 0.01), but still far from the amounts of gliding normally displayed in 50 μM of AITC (11.6 ± 3.8%, *n* = 11, *p* < 0.001). Scrunching behaviors were also higher than their no-AITC control values (21.1 ± 6.6%, *n* = 9, *p* < 0.01), but also still far from the amounts of scrunching displayed in 50 μM of AITC (83.4 ± 4.2%, *n* = 11, *p* < 0.001). At days 71 and 85, both gliding (respectively 27.5 ± 9.0%, *n* = 10, *p* = 0.13 and 25.7 ± 7.1%, *n* = 12, *p* = 0.052) and scrunching (respectively 69.4 ± 9.0%, *n* = 10, *p* = 0.68 and 70.6 ± 6.9%, *n* = 12, *p* = 0.77) reached behaviors levels that were significantly similar to their 50 μM AITC controls.

### 3.2 Behavior (1x feeding)

Worms fed TRPA1 dsRNA once displayed similar behavior trends than those fed three times (Figures 2A,B). Still, at day 7, gliding behaviors were significantly lower than the no-AITC control (74.1 ± 7.8, *n* = 11, *p* < 0.01) and scrunching behaviors were higher (16.4 ± 8.0, *n* = 11, *p* < 0.05). This may be the sign of the progressive loss of phenotype. At day 21, both gliding and scrunching behaviors reached levels similar to the no-AITC controls (respectively 94.0 ± 3.5%, *n* = 12, *p* = 0.75 and 0.0 ± 0.0, *n* = 12). This held true at day 35, with gliding levels at 91.7 ± 5.9% (*n* = 11, *p* = 0.78) and scrunching levels at 6.0 ± 6.0 (*n* = 11, *p* = 0.54). At day 49, gliding levels seemed to decrease, but were not significantly different (77.5 ± 8.0, *n* = 12, *p* = 0.09), although scrunching levels slightly but significantly increased (17.5 ± 7.0%, *n* = 12, *p* < 0.05). At day 63, both gliding and scrunching were significantly different then the no-AITC control (respectively 68.1 ± 5.7%, *n* = 12, *p* < 0.01 and 21.4 ± 4.8%, *n* = 12, *p* < 0.01). At day 77, both gliding and scrunching reached levels comparable to controls in 50 μM. Gliding behaviors reached 5.6 ± 3.3% (*n* = 11, *p* < 0.05), which surprisingly was even significantly lower than the control, and scrunching behaviors reached 91.2 ± 3.6 (*n* = 11, *p* = 0.08), no different than the control.

### 3.3 Behavior comparisons

To compare the effects on behavior of the two feeding schedules (1x vs. 3x), two options were possible: we could either compare behavior on days after the first or the last feeding. Because feeding the animals three times takes one more week than feeding them only once, the two feeding timelines do not match when compared on days after first RNAi feeding. However, as we expect behavior to be correlated to the amount of gene expression, and because the first feeding might be efficient enough to reduce said gene expression, we still chose to mainly analyze the behavior on days after first feeding together (Figures 2A,B). This option was best to display the progressive loss of phenotype and to represent both methodologies in real time. The second option, plotting behaviors by days after last feeding, was best to compare the retrieval of phenotype between the two feeding methods and is available as Supplementary Figure 1. This plot shows similar trends between 1x and 3x feedings with an exception at day 63 where animals fed once seem to show less nociceptive behaviors, suggesting that the retrieval of normal phenotypes took more time. This was unexpected, as we would expect supplementary feedings to induce longer-lasting effects.

### 3.4 GFP controls

To ensure that the RNAi methodology we were using did not influence planarians’ behavior in the long run, we also fed the worms with GFP dsRNA. Because planarians do not express GFP endogenously, feeding them GFP dsRNA should not induce any change in gene expression, nor in behavior. Planarians were fed GFP dsRNA three times over the course of a week and were then exposed to 50 μM of AITC after 1, 3 and 5 weeks (Figure 2C). Without any RNAi feeding, when exposed to 50 μM of AITC, planarians display little gliding (11.6 ± 3.8%, *n* = 11) and mostly scrunching (83.4 ± 4.2%, *n* = 11). When fed GFP dsRNA, the AITC-exposed worms display the same amount of gliding over time, without any significant change (day 7: 19.0 ± 4.8%, *n* = 14, *p* = 0.17; day 21: 24.4 ± 11.2%, *n* = 5, *p* = 0.11; day 35: 22.3 ± 12.0%, *n* = 8, *p* = 0.74). Scrunching behaviors also stay stable over 1 week (79.9 ± 5.1%, *n* = 14, *p* = 0.83) and 3 weeks (68.8 ± 13.0%, *n* = 5, *p* = 0.28). After 5 weeks, scrunching behaviors were significantly lower (58.1 ± 10.2%, *n* = 8, *p* < 0.05). However this reduction of scrunching behaviors was not compensated for by an increase in non-nociceptive gliding behaviors. Instead, we observed an increase in ‘other’ behaviors (control: 5.0 ± 2.2%, *n* = 11; day 7: 1.1 ± 0.6%, *n* = 14, *p* = 0.21; day 21: 6.8 ± 2.4%, *n* = 5, *p* = 0.45; day 35: 19.6 ± 6.3%, *n* = 8, *p* < 0.05). ‘Other’ behaviors include everything that is not either gliding or scrunching. Usually, these behaviors appeared to be head tilts, twitching, sudden change of direction, twisting, etc. They tend to be ‘uncomfortable’ behaviors, which could also be interpreted as nociceptive. Hence, our interpretation is that the reduction in scrunching behaviors does not necessarily imply a change in behavior induced by the RNAi feeding. Instead, it is rather the smaller number of animals used in these conditions and the resulting bigger standard deviation that created this statistical difference. Furthermore, even if the amount of scrunching behavior was indeed slightly lower 5 weeks after a GFP dsRNA ingestion, it was still highly significantly different from the TRPA1 RNAi groups (60% against 0%). Most importantly, gliding behaviors did not significantly differ throughout the weeks, so we assumed that our RNAi methodology controls did not induce significant behavioral changes.

### 3.5 RT-qPCR

Lastly, we monitored endogenous TRPA1 expression in planarians fed only once throughout the weeks (Figure 2D). We first submitted the three candidate HKGs GAPDH, EF2, and 18S to RefFinder analysis in order to determine the best HKG to normalize TRPA1 endogenous expression in planarians fed only once with TRPA1 dsRNA. The ranking and stability values for each HKG are listed in Table 2. GAPDH was identified as the most stable and therefore the best reference gene with NormFinder and with the comparative Δ-Ct method. GeNorm identified GAPDH/18S as the best pair of reference genes. According to Bestkeeper, 18S had the lowest standard deviation and the best Pearson coefficient of correlation, meaning that its expression is the most stable and is well correlated to the pattern of the two other reference genes. By combining the results obtained with these different algorithms, RefFinder identified GAPDH as the best reference gene. TRPA1 relative expression has therefore been normalized by GAPDH (Figure 2D). The graph showing TRPA1 expression normalized by 18S and EF2 are shown in Supplementary Figure 2 and exhibits the same expression pattern as when normalized by GAPDH. As depicted in Figure 2D, the relative level of *Gd*-TRPA1 mRNA expression 1 week after the dsRNA feeding dropped drastically from 100 ± 14.9% (*n* = 7) to 11.3 ± 1.7% (*n* = 6, *p* < 0.01). Levels slowly increased again throughout the following weeks, reaching 25.4 ± 6.9% (*n* = 6, *p* < 0.01) after 49 days. At 56 days, RNA levels reached 54.6 ± 11.7% (*n* = 6), which was not significantly different than the control anymore (*p* = 0.17). However, expression levels did not fully reach control levels yet, as at 77 days mRNA levels were still significantly different from the control at 41.9 ± 6.4% (*n* = 6, *p* < 0.05).

**Table 2:** RefFinder analysis of housekeeping gene stability and ranking.

NormFinder		GeNorm		BestKeeper		
Reference Gene	Stability value	Reference Gene	Stability value (M)	Reference Gene	Standard deviation	Pearson correlation coefficient (r)
GAPDH	0.416	GAPDH/18S	0.680	18S	0.91	0.965
18S	0.538	EF2	0.724	EF2	1.01	0.909
EF2	0.571			GAPDH	1.12	0.941

Δ-Ct method		Comprehensive ranking	
Reference Gene	Average standard deviation	Reference Gene	Geomean ranking values
GAPDH	0.69	GAPDH	1.32
18S	0.73	18S	1.41
EF2	0.75	EF2	2.71

The NormFinder algorithm calculates a stability value number for each gene, lower values indicating higher stability. GeNorm calculates a stability value (M) via pairwise comparisons and determines the best pair of reference genes, lower M values indicating greater stability. BestKeeper determines a standard deviation and a Pearson coefficient of correlation (r), and determines the best reference gene based on these two variables. Lower standard deviation values indicates the best expression stability while values of r closer to 1 indicates the best correlation to the expression of the other HKGs. The Δ-Ct method compares relative expression of pairs of genes within each sample, lower standard deviation values indicating greater stability. Based on the rankings from each algorithm, RefFinder assigns an appropriate weight to an individual gene and calculates the geometric mean of their weights for the overall final ranking.

## 4 Discussion

### 4.1 Target specificity

In 2013, Rouhana et al. already showed by *in situ* hybridization that one feeding was sufficient enough to induce significant and long-lasting decrease of mRNA expression (Rouhana et al., 2013). They also demonstrated that the amount of mRNA expression decrease is gene specific. This is expected, as it relies upon many factors: (i) the amount of mRNA expressed for a given gene, (ii) its correlated amount of proteins at a given time point, (iii) the mRNA and protein half-lives (during the loss of phenotype), and (iv) the protein synthesis rate (during the phenotype retrieval). Hence, we cannot extrapolate the timeline of this current study to each and every gene of interest. Proteins expressed in great quantities and with a very long turnover would imply late phenotypic manifestations, as already mentioned by Rouhana et al. (2013), and would thus need multiple or even continuous dsRNA treatments. However, as previously mentioned, a few studies have already successfully knocked protein expression down by only feeding or injecting dsRNA once, thus suggesting that it might be suitable for most protocols. For example, Sánchez Alvarado and Newmark micro-injected myosin dsRNA only once and looked at the body-wall musculature in *Smed* (Sánchez Alvarado and Newmark, 1999); Rouhana et al. fed *Ago2* dsRNA (a component of the RISC complex involved in RNA silencing) to *Smed* planarians and observed lethality 19 days after only one feeding (Rouhana et al., 2013); Cochet-Escartin et al. also fed SLO1 dsRNA, a gene responsible for the production of calcium-activated big potassium (BK) channels, and observed ethanol-dependent behavioral outputs in *Smed*, although it is not clear if they fed them once or multiple times (Cochet-Escartin et al., 2016); finally, in this present study, we also showed that one single feeding of *Gd*-TRPA1 dsRNA was enough to produce similar behavioral outputs as with three feedings in *Gd* after 1 to 2 weeks. This is consistent with the fact that most mammalian proteins have a half-life of around 1 week (Mathieson et al., 2018; Rolfs et al., 2021). In invertebrates, protein turnover might be longer (Hawkins, 1991), but a recent study by Lee et al. showed that the turnover of planarian (*Schmidtea mediterranea*) epidermal cells was averaging 5 days (Lee et al., 2024), which coincides with the loss of nociceptive phenotypes observed in this present study.

### 4.2 Nociceptive behaviors

Behavior assessments (Figures 2A,B) showed that the nociceptive phenotypes were only partially abolished 1 week after the first feeding. This may be explained by the persistence of proteins that are not yet degraded. To get the full knockdown potential of this RNAi methodology, we would recommend waiting at least 2 weeks after the start of the RNAi protocol to start behavioral tests. This would mean that feeding the animals only once requires less materials but does not save time compared to feeding them multiple times in the span of a week. However, even though nociceptive behaviors were not fully reduced after 1 week, they were still highly significantly different than the control group (approx. 80% of total effect), which is what is expected from this sort of protocol. Hence, if a slight increase in efficiency of the RNA interference over time is not an issue, then behavioral tests could as well be done 1 week after one unique dsRNA treatment.

### 4.3 TRPA1 expression

As we have seen in our previous study (Reho et al., 2024), the amount of both exogenous dsRNA and endogenous *Gd*-TRPA1 mRNA levels were already at their respective maximum and minimum 4 days after the first feeding. Feeding the worms dsRNA two more times did not further increase the amount of exogenous dsRNA present in the animal, nor did it further reduce the amount of endogenous *Gd*-TRPA1 mRNA expression. In (Sabry et al., 2019), TRPA1 expression was reduced by 51 to 73% after four injections in *Schmidtea mediterranea (Sm)* or *Dugesia japonica (Dj)*. In (Arenas et al., 2017), feeding *Sm* worms three times with dsRNA reduced Smed-TRPA1 mRNA levels by 50%. In (Inoue et al., 2014), TRPAa expression was reduced by more than 80% after three feedings in *Dj*. In this present study, *Gd*-TRPA1 mRNA expression was already reduced by more than 85% one week after a unique treatment and it slowly increased again throughout the following 7 weeks (Figure 2D), demonstrating that we achieved similar knockdown efficiency for the same gene with only one single dsRNA treatment. As for the retrieval of mRNA expression, while still significantly different from the control values, the mRNA expression of *Gd*-TRPA1 already reached back more than 40% after 11 weeks, which seemed to be sufficient for the retrieval of the scrunching phenotype. The fact that these final values are still rather low either suggest that our control group contains high expression values (it does not, however, contain any outlier data); or that the retrieval of mRNA expression is indeed longer than 11 weeks.

## 5 Conclusion

In the light of the 3 Rs, refining protocols that can take several weeks to set up is a valuable way to save time, resources and animals. In this study, we demonstrated that, while most articles available in the literature feed planarians dsRNA at least three times, feeding them only once with an appropriate amount of dsRNA might just be enough to induce similar reductions of most genes expression and to induce similar behavioral changes.

## Data Availability

The original contributions presented in the study are included in the article/Supplementary material, further inquiries can be directed to the corresponding author.
