# Supplementary material for: Planarian RNAi knockdown: feeding once might just be enough
Source: Front Neurosci. 2025 Apr 30;19:1546196. doi: 10.3389/fnins.2025.1546196 (PMC12075233; doi:10.3389/fnins.2025.1546196)
Supplement: Supplementary file 1 [file Data_Sheet_1.ZIP › Reho et al - RNAi refinement supplementary figures.pdf]

Supplementary Material

1 Supplementary Data

All raw tracking and behavioral data and the source code for statistical analyses and graphical plots are included as supplementary materials.

2 Supplementary Figures

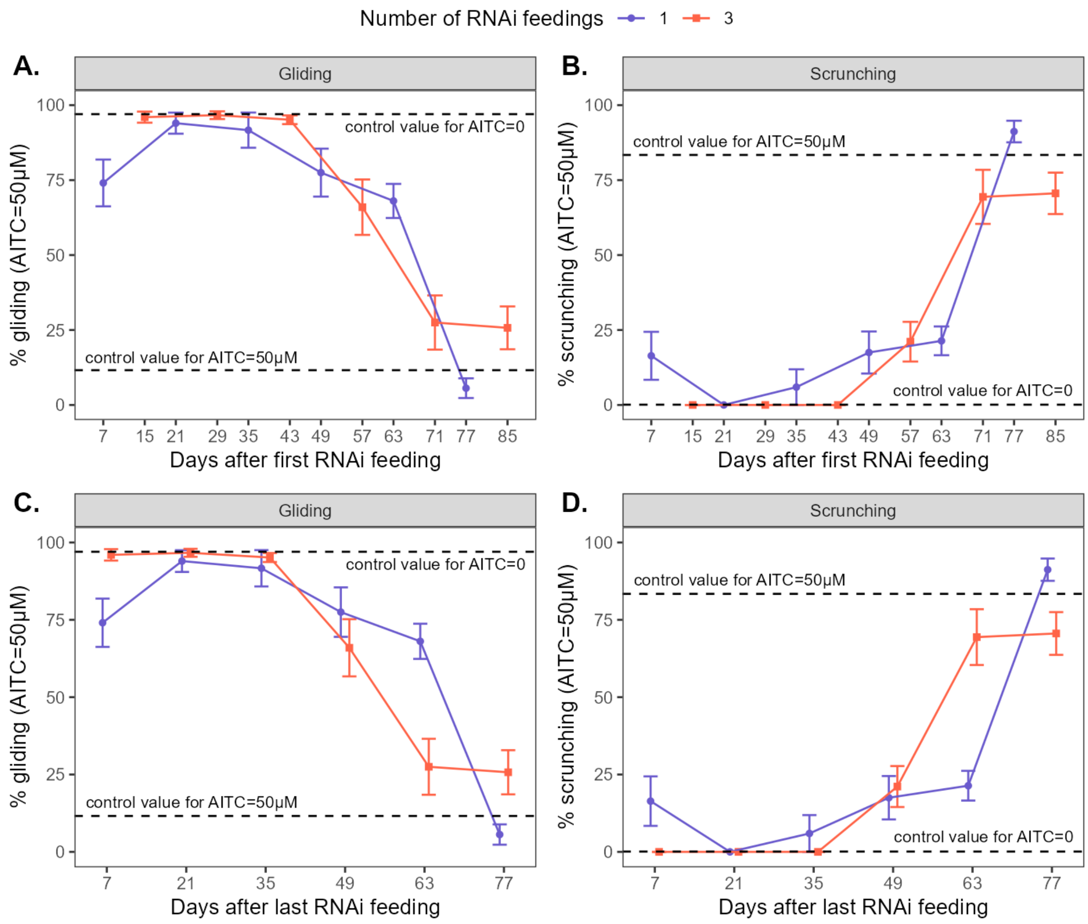

Supplementary Figure 1. Gliding and scrunching behaviors of planarians observed when exposed to 50 µM of AITC for 5 minutes every other week after being fed *Gd*-TRPA1 dsRNA. Panels **A** and **B** align days after the first day of feeding (3 RNAi feedings offsets the time course by a week, useful to compare the loss of phenotype). Panels **C** and **D** align days after the last day of feeding (useful to compare the retrieval of phenotype). Dots represent the mean, and error bars represent the SEM. Dashed lines represent the mean value for untreated worms exposed to either no AITC (plain mineral water) or 50 µM AITC.

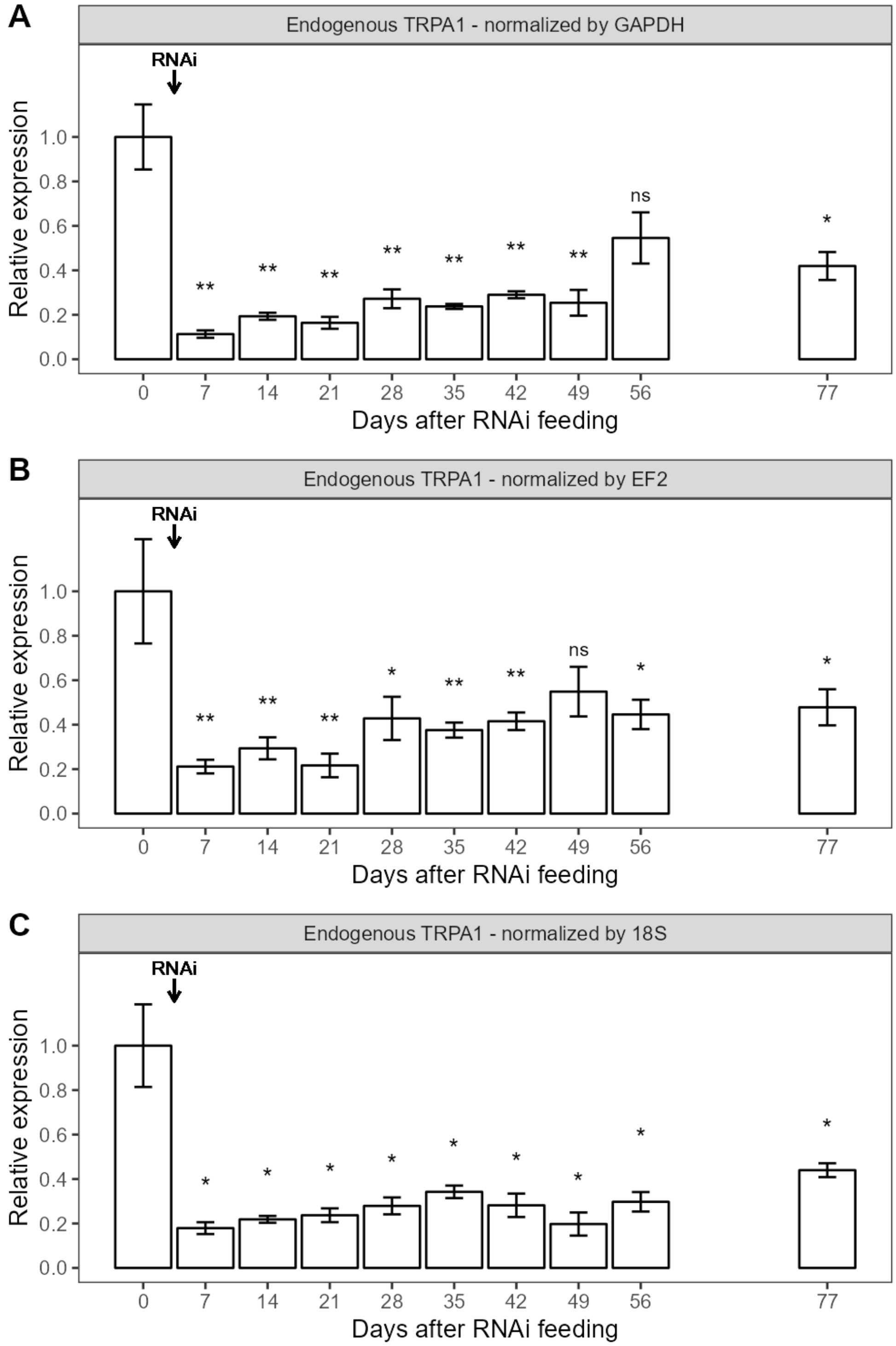

Supplementary Figure 2. Relative expression of *Gd*-TRPA1 throughout the entire experiment normalized by 3 different house keeping genes (HKGs). As discussed in the manuscript, the software RefFinder suggested that GAPDH **(A)** was the best candidate reference gene, but we provide here the additional HKG results, Elongation Factor 2 **(B)** and 18S ribosomal RNA **(C)**, as means of comparison.
